# The Antimicrobial Properties of Cedar Leaf (*Thuja plicata*) Oil; A Safe and Efficient Decontamination Agent for Buildings

**DOI:** 10.3390/ijerph8124477

**Published:** 2011-11-30

**Authors:** James Hudson, Michael Kuo, Selvarani Vimalanathan

**Affiliations:** Department of Pathology & Laboratory Medicine, University of British Columbia, 2733 Heather Street, Vancouver, BC V5Z 3J5, Canada; Email: mkuo1@sfu.ca (M.K.); vnathanm@telus.net (S.V.)

**Keywords:** anti-microbial, decontamination, sick building syndrome, cedar leaf oil, *Thuja plicata*, bacteria, fungi, spores

## Abstract

Cedar leaf oil (CLO), derived from the Western red cedar, *Thuja plicata*, was evaluated as a safe and acceptable broad spectrum antimicrobial agent, with a view to its potential applications in buildings, including the alleviation of sick building syndrome. Various Gram-positive and Gram-negative human bacteria, and two fungal organisms, all known to be common environmental sources of potential infection, were selected and tested quantitatively, and all of them were found to be susceptible to CLO liquid and vapor. Bacterial spores and *Aspergillus niger* were sensitive, although less so than the vegetative bacteria. Similar tests with cultured human lung cells showed that continuous exposure to CLO vapor for at least 60 minutes was not toxic to the cells. Based on these results, CLO shows promise as a prospective safe, green, broad-spectrum anti-microbial agent for decontamination of buildings.

## 1. Introduction

The buildings we inhabit and work in have become the focus of increased attention recently from the health point of view, because of the realization that the air within the buildings is a potential source of pathogenic micro-organisms and their toxins. These may be introduced into a building by people themselves or through the ventilation systems, with ensuing sickness. This has led to the concept of “sick building syndrome”, which is characterized by the inhabitants complaining of various symptoms, including headaches, nausea, fevers, exacerbation of asthma and allergic reactions [1,2,3]. Several fungal and bacterial species have been incriminated as sources of toxins and volatile compounds [4,5], but in addition other introduced environmental and human pathogenic microbes may result in infections [6,7].

One solution to these problems, which would circumvent the necessity of costly renovations [8], could be periodic treatment of rooms and ventilation ducts with a broad-spectrum antimicrobial agent. However most of the products on the market are themselves toxic and can only be used safely in empty buildings [5,9,10,11,12].

Many essential oils also possess antimicrobial properties [13,14,15,16], including oil derived from European species of *Thuja* [17], although many of them would not be considered safe or economical for a large scale operation. However, the oil of Western red cedar leaves (*Thuja plicata*) has been used traditionally among Aboriginal peoples of the Pacific north west to treat a variety of upper respiratory symptoms and wounds. The relatively mild odor is considered to be safe, pleasant and acceptable. Based on these observations a pilot study was carried out in an office building, which was exposed to vaporized cedar leaf oil delivered through the ventilation system, and random swab samples of various surfaces before and after the treatment were compared for bacterial and fungal counts. The results indicated a substantial reduction in microbial load. We therefore decided to evaluate this product quantitatively, as a liquid oil and vapor, for anti-bacterial and anti-fungal activities against a number of known pathogenic and environmental organisms. We also included two other bacteria which are important pathogens in the veterinary field. The objectives were: to evaluate the efficacy of CLO and CLO-vapor against selected organisms and spores; and to determine that cultured human cells do not suffer cytotoxic effects from exposure to CLO vapor.

## 2. Experimental Section

### 2.1. Materials

The cedar-leaf oil was obtained from Tree of Life Essential Oil, Port Hardy, BC, Canada. The product was obtained by steam distillation of *Thuja plicata* (Western red cedar) leaves, and contained >80% thujone.

All the test organisms were ATCC strains, obtained from PML Microbiologicals, Oregon. The bacteria, *Bacillus subtilis*, *Streptococcus pyogenes*, *Hemophilus influenzae*, *Acinetobacter baumannii*, *Enterococcus fecalis*, *Salmonella enteritidis*, and *Escherichia coli*, were propagated and assayed, as colony-forming units (CFU), in Mueller-Hinton broth and agar plates. *Candida albicans* and *Aspergillus niger*, also ATCC strains, were maintained and assayed on Sabouraud-dextrose agar plates. Human lung epithelial cells (A549), were obtained from ATCC, and propagated in flasks in Dulbecco MEM medium supplemented with 5% fetal bovine serum, without anti-microbial agents, in a 5% CO_2_ incubator at 37 °C.

### 2.2. Methods

Bacterial working stocks were obtained for each experiment by selecting 4–6 colonies from an agar plate and growing them in Mueller-Hinton broth. Cells were pelleted by centrifugation, washed and resuspended in PBS (phosphate buffered saline) to approximately 10^6^ cfu/mL. For the reactions with liquid CLO 20 or 100 µL of diluted bacteria were added to replicate Eppendorf tubes and mixed with the appropriate volume of CLO, at ambient temperature (22 °C). Individual tubes were taken at various times and the bacteria were pelleted in a microfuge, washed with PBS and assayed for cfu on agar plates. The centrifugation time in the microfuge was less than a minute; consequently this was not a significant addition to the exposure time during the reactions. Controls consisted of identical numbers of bacteria mixed with phosphate buffered saline (PBS) and processed in the same way without exposure to CLO. In some experiments CLO was replaced by tea tree oil (TTO) as a positive control. In experiments to evaluate the possible effect of light on the anti-bacterial activity, half of the Eppendorf reaction tubes were completely enclosed in aluminum foil to preclude ambient light (dark reactions [18]).

Dried films of bacteria were obtained by placing 5 µL of the stock bacterial suspension on the inner side of an Eppendorf tube cap and allowing to dry. A separate Eppendorf tube was then used for the oil “reservoir”, onto which the cap with dried bacteria was inverted. After an appropriate exposure time the bacteria were reconstituted by washing them off the cap with PBS, pelleted and washed, followed by assay for cfu. In some experiments 50 µL aliquots of bacteria were dried onto glass slides, which were then inverted over a jar containing the oil, followed by reconstitution in PBS, washing and assaying for cfu. In all experiments, only a fraction of the CLO actually evaporated during the reactions; thus continuous exposure to the dried films was obtained for many hours without replenishing the oil, an observation that is relevant to proposed use in the field.

Endospores of *B. subtilis* were prepared by growth of the bacteria on agar plates, followed by induction of spore formation by continued incubation with the plates sealed in parafilm. This was followed by rinsing off the upper spore layer, and treatment with distilled water at 80 °C [16]. Further purification was achieved by the histodenz centrifugation method described by Sorg and Dineen [19]. The pellet obtained with 45% histodenz was essentially free of vegetative/dead cells and debris, as determined by malachite green-safranin staining.

*Candida albicans* yeast cells were grown in Sabouraud-dextrose broth and treated in an analogous fashion to the bacteria, in liquid and dry film reactions, followed by assay for cfu on Sabouraud-dextrose (SB) agar plates. Aspergillus niger was grown on SB plates, and samples were removed for resuspension in PBS. Dried films were prepared as described above, and these were treated and assayed by inspection for growth or no growth on SB plates.

Cell viability was measured on monolayers of human lung A549 cells grown to confluence in 6-well trays. For treatment the media were removed by aspiration, and the moist cells were exposed for various times to CLO vapor. Following a further 24 h incubation in normal medium, cell viability was measured by the lactate dehydrogenase (LDH) method, using the kit obtained from Sciencell (Carlsbad, CA, USA), and following the instructions provided. The final assay reactions were measured in a micro-plate reader at a wavelength of 450 nm.

## 3. Results

### 3.1. Antibacterial Tests with Liquid CLO

A total of seven bacteria, known to be potentially pathogenic to humans and recognized as environmental contaminants, were selected for evaluation. It was found that most of the materials used routinely in the laboratory were appropriate for tests (*i.e.*, they were resistant to CLO liquid and vapor), with the exception of polystyrene culture vessels (trays, tubes, flasks), which were degraded by direct contact with CLO. The resulting degradation product was toxic to bacteria and to cultured cells. Consequently polypropylene and glass vessels were used in the experiments. Other materials tested, such as metals, wood, hard plastic, glass and fabrics, appeared to be resistant to CLO. 

**Figure 1:**
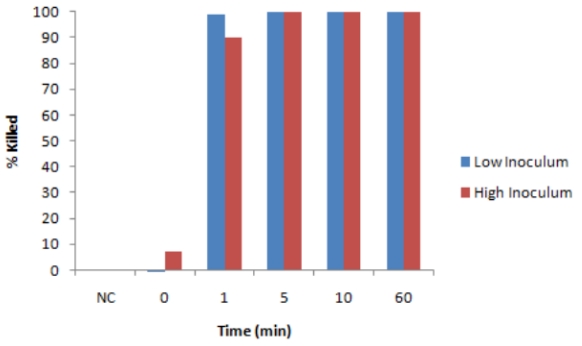
Antibacterial effect of CLO: Variation in bacterial number. Reaction mixtures (100 µL) contained either 1,500 cfu or 150,000 cfu of *B. subtilis* in saline with 1% CLO. At various times reactions were stopped by pelleting the bacteria in Eppendorf tubes, and the latter were washed in saline and assayed for cfu. Blue bars: low inoculum; red bars: high inoculum. % kill in comparison with unexposed controls. NC, untreated control.

**Figure 2:**
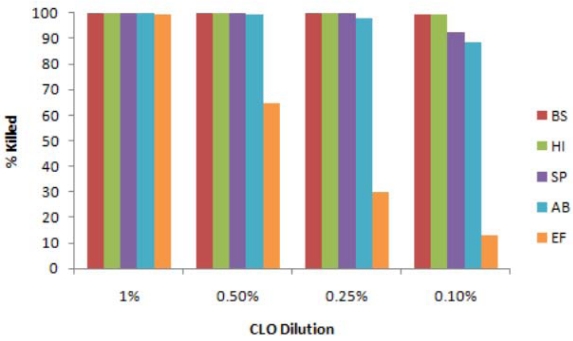
Effect of CLO against different bacteria. Reactions, containing 1,500 cfu of each bacterium in 100 µL of saline and the indicated concentration of CLO, were incubated at 22 °C for 60 min. Bacteria were then pelleted by centrifugation, washed and assayed for cfu, in comparison with unexposed control bacteria. BS = *B. subtilis*, HI = *H. influenzae*, SP = *S. pyogenes*, AB = *A. baumannii*, EF = *E. fecalis*.

Initial tests were carried out with *B. subtilis*, using TTO, a well-known antibacterial oil [13] as a positive control, and an equivalent number of unexposed bacteria as a negative control. The antibacterial efficacy was affected by time of exposure and concentration of CLO, as expected, but was not significantly influenced by the number of bacteria, as shown in Figure 1. This latter point was important to establish since the number of viable organisms in the field are likely to vary considerably from a few bacteria to many thousands. *B. subtilis* was readily killed by CLO after several minutes contact with the oil (Figure 1). Concentrations of CLO down to 0.1% (in saline) completely killed the bacteria within one hour (Figure 1 and Figure 2). Further dilutions showed successively less efficacy. 

Four of the other bacteria were tested by the same protocols, and all of them were readily killed by CLO down to high dilutions, as shown in Figure 2, although the relative sensitivities varied. *H. influenzae* (Gram –) and *S. pyogenes* (Gram +) were as susceptible as *B. subtilis* (Gram +), whereas *A. baumannii* (Gram –) was slightly more resistant, and *E. fecalis* (Gram +) was substantially more resistant. Nevertheless even the latter was completely killed by exposure to 1% CLO. In addition *S. enteritidis* (Gram –) was very sensitive, but *E. coli* (Gram –) was slightly more resistant (data not shown). To determine if these activities of CLO were bactericidal or bacteriostatic, representative treated culture plates were returned to the incubator for several days, but no growth or additional growth occurred. In addition when the treated “bacteria” were pelleted by centrifugation, washed in saline and resuspended in broth, no growth was observed. Thus the activities were bactericidal.

Since CLO is immiscible with aqueous solutions, incubations of CLO with bacterial suspensions required frequent agitation or mixing, although efficient bacterial killing was achieved. Attempts were made to improve the stability of the emulsions by incorporating small amounts of the neutral detergent Tween 80 into the oil, as recommended by studies on Taxandria oil [20]. However the addition of either Tween 80 or Tween 20 did not improve the antibacterial efficiency of CLO any further and consequently they were omitted in subsequent tests.

### 3.2. Possible Effect of Light

**Figure 3:**
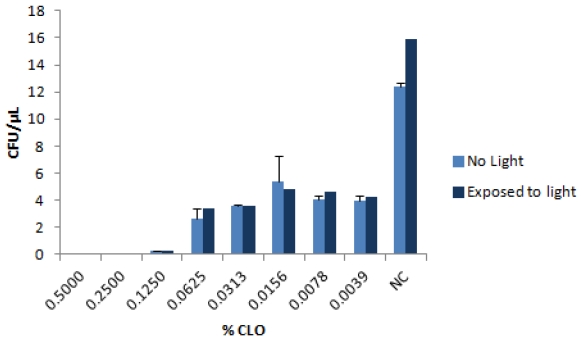
Effect of light exposure on antibacterial property of CLO. Reactions contained 1,500 cfu of *B. subtilis* and the indicated concentration of CLO. Half the reactions were exposed to fluorescent light in the biosafety cabinet, and the others were completely covered in aluminum foil to exclude light. Reactions were processed and assayed for cfu as usual, in comparison to the untreated control (NC).

Some plant-based antimicrobials are influenced by ambient light. In theory the antibacterial activity could be enhanced by light (due to the presence of photosensitizers, [18]), or reduced as a result of photo-degradation. Since the CLO applications in buildings could take place in either the presence or absence of ambient light, it was important to establish that the efficacy of the CLO would not be affected significantly. This was tested with *B. subtilis*, but the efficacy of killing by CLO was essentially the same in light or dark, as shown in Figure 3.

### 3.3. Sporicidal Activity of CLO

Conventional methods of producing spores for study often contain mixtures of spores and vegetative cells. In order to ensure that prospective antimicrobials are really effective against spores, it is desirable to experiment with preparations that are relatively free of dead and vegetative forms [10]. Recently a method of purifying spores was reported for *Clostridium difficile* (an anaerobic intestinal pathogen), which involved centrifugation of partly purified spores through gradients of Histodenz [19]. The spores of *C. difficile* sedimented through 50% Histodenz, leaving them free from the vegetative cells and dead organisms, which were found in the layers of less dense Histodenz.

In our experiments with different concentrations of Histodenz, it was shown that purification of *B. subtilis* spores worked best by sedimentation through 45% Histodenz (they did not sediment through 50%). This purified spore preparation was tested in a time course experiment, and the results are summarised in Table 1. Increasing exposure times and concentrations resulted in greater killing of the spores, although the rate and level of killing achieved was not as great as that observed with the vegetative *B. subtilis*.

**Table 1:** Sporicidal activity of CLO; % kill (cfu).

Exposure time hours	1% CLO	5% CLO	10% CLO
24	30.5	48.0	63.6
48	41.2	50.0	88.0
72	64.5	70.2	93.4

### 3.4. Antibacterial Effects of CLO Vapor on Dried Films

In subsequent experiments the bacteria were used as dried films on glass slides, to represent normal conditions of surface contamination. The treated films were reconstituted in saline solution after exposure, followed by serial dilution and measurement of CFUs on agar plates. The films, containing different numbers of viable bacteria, were found to be just as vulnerable to liquid CLO as bacterial suspensions (data not shown).

**Figure 4:**
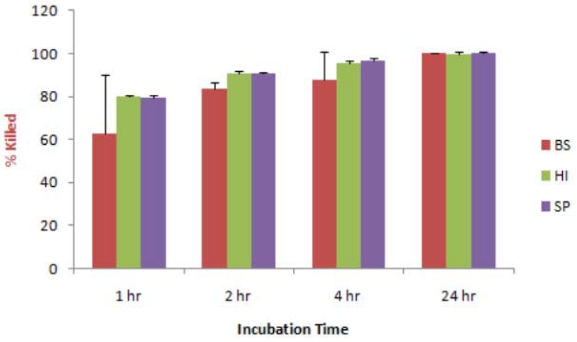
Effect of CLO-vapor on dried bacteria. 100 µL each bacterium were air dried on glass slides, and exposed to the vapor of CLO in a glass vessel, at 22 ºC. The bacteria were then reconstituted in saline and assayed for CFU, in comparison with unexposed bacteria. BS = *B. subtilis*, HI = *H. influenzae*, SP = *S. pyogenes.*

The next series of experiments consisted of exposing similar dried films of bacteria to the vapor of CLO. This treatment was also very effective, although the time of exposure required for efficient killing was significantly longer than with the liquid oil. Results are shown in Figure 4 for the three bacteria, *B. subtilis*, *H. influenzae*, and *S. pyogenes*. In separate tests dried films of *A. baumannii* showed a similar level of susceptibility.

### 3.5. Effect of CLO on *Candida albicans*

The pathogenic yeast *C. albicans* was exposed to CLO liquid or vapor and assayed on agar plates as CFU (analogous to bacterial cfu). The organism was readily killed by liquid CLO, with a sensitivity similar to *B. subtilis* and *H. influenzae* (data not shown). The results in Table 2 show that this organism is as susceptible to CLO vapor as the bacteria.

**Table 2:** Activity of CLO vapor against *Candida albicans*.

Exposure time hours	*C. albicans* (cfu)	% kill
0	1,270 ± 58	
4	67 ± 3.4	95
24	0	>99.9

### 3.6. Effect of CLO-Vapor on *Aspergillus niger*

The fungus (mold) *A. niger* is a common environmental organism which can cause serious disease in humans with compromised immune systems. It can be grown and assayed conveniently on appropriate agar plates, although it tends to produce filamentous growth rather than discrete colonies. Quantitative measurements can be performed by serial dilutions and determination of growth end-point. The results indicated that this organism is also susceptible to CLO-vapor, although at this time we cannot be sure if it is irreversibly killed (fungicidal) or just growth-inhibited (fungistatic), since a few colonies did eventually appear on plates of treated *A. niger* following prolonged cultivation. Nevertheless even if eradication was not achieved, the prospect of its control by CLO vapor is realistic.

### 3.7. Cytotoxicity of CLO Vapor

It is important to establish experimentally that short-term exposure to CLO vapor is harmless to building occupants and operators. This was evaluated by exposing cultures of human lung cells (A549 epithelial cell line), with culture medium removed to permit exposure of the monolayer of cells, to CLO vapor for different times. This was followed by microscopic examination of the cells for signs of cell toxicity or death, and assays for cell viability. No signs of cytopathology were evident, and the results of the LDH (lactate dehydrogenase) assay (Table 3) showed that exposures up to 60 minutes had no deleterious effects on the cells. 

**Table 3:** Viability of cells exposed to CLO vapor.

Time of exposure to CLO vapor (min)	Cell survival% of control
10	97.5 ± 13.7
30	96.7 ± 7.6
60	97.0 ± 8.3

## 4. Discussion

The causes of sick building syndrome have been ascribed to a number of agents, including volatile organic compounds (VOC’s) and micro-organisms [2,3]. The latter could be responsible for causing infections and/or inflammatory responses in inhabitants, leading to exacerbation of asthmatic and allergic conditions [7,21,22], Many broad-spectrum chemicals have been advocated as microbial decontaminants in various situations, but because of safety concerns these materials are normally restricted to applications in empty rooms or buildings [11,12]. In contrast several essential oils with anti-bacterial activities could be more acceptable [15,17,23,24], although they might not be economically feasible for large-scale applications. Western red Cedar leaf oil (CLO) circumvents some of the hazard and economical issues, because of its known traditional uses among the Aboriginal peoples of the Pacific northwest, and some promising preliminary tests in local office buildings. Furthermore CLO presents a ”green” solution to the problem since it is produced simply and economically from cedar branches without damaging the trees. These considerations prompted the laboratory studies reported here. 

All the organisms selected for testing (seven bacteria, bacterial spores, and two fungi), representing human pathogens commonly encountered in the environment, were susceptible to CLO. The 7 bacteria consisted of three Gram-positive organisms, *Bacillus subtilis*, *Streptococcus pyogenes*, and *Enterococcus fecalis*, and four Gram-negative organisms, *Acinetobacter baumannii*, *Hemophilus influenzae, Salmonella enteritidis*, and * Escherichia coli*. They were all readily killed by CLO, although their relative sensitivities were somewhat different. These activities were bactericidal rather than bacteriostatic. Purified spores of *Bacillus subtilis* were also sensitive to CLO, but they were relatively resistant in comparison to the corresponding vegetative (growing) cells. In general bacterial spores were found to be much more resistant than vegetative cells to a variety of antibacterial agents and disinfectants [10,25]. In connection with the issue of organic load in the microbial samples, recent preliminary tests have shown that the antibacterial activity of CLO was not measurably affected by the inclusion of 10% fetal bovine serum (cell culture grade) (data not shown). Nevertheless, further study on possible effects of different organic loads should be carried out.

Two fungi, the medically important yeast *Candida albicans*, and the filamentous mold *Aspergillus niger*, and were also readily inactivated by CLO. *C. albicans* was as sensitive as the more sensitive bacteria, although the *A. niger* appeared to recover to some degree after several days of incubation. Further studies are required to determine if the activity against *A. niger* is fungicidal or fungistatic. However even if CLO cannot completely eradicate the fungus, its ability to curtail fungal growth would still be beneficial.

Human lung cell cultures did not show any signs of cytotoxicity following short-term exposure to CLO vapor. Since lung and oral epithelial cells would be the first cells to encounter CLO vapor during exposure, this result supports the belief that CLO is safe to use in buildings in the presence of people, a significant advantage over many other decontamination agents.

The mechanism of action of CLO is difficult to determine at present, as there are clearly several variables involved. The Gram-staining status of the organisms was not a discriminating factor, since this did not affect the relative susceptibility of the organism to CLO. The *Thuja* oils generally contain more than 30 different volatile compounds, and although the major component thujone does by itself possess various pharmacological activities, studies with other oils indicated that the entire oil is not necessarily equivalent to a single component [17,23]. This should not be surprising in consideration of the well known synergistic biological properties of many plant-derived medicines [26]. Furthermore the vapor might not always be chemically and biologically equivalent to the liquid form of the oil [24], and this would introduce another variable. In conclusion, these results show that CLO holds promise as a prospective safe, green, broad-spectrum, anti-microbial agent for decontamination of buildings.
